# Pharmacists' Knowledge and Practice of Issues Related to Using Psychotropic Medication in Elderly People in Ethiopia: A Prospective Cross-Sectional Study

**DOI:** 10.1155/2020/7695692

**Published:** 2020-08-12

**Authors:** Gashaw Binega Mekonnen, Alemante Tafese Beyna

**Affiliations:** ^1^College of Medicine and Health Science, School of Pharmacy, Department of Clinical Pharmacy, University of Gondar, P.O. Box-196, Gondar, Ethiopia; ^2^College of Medicine and Health Sciences, School of Pharmacy, Department of Pharmacology, University of Gondar, Gondar, Ethiopia

## Abstract

**Purpose:**

This study is aimed at assessing pharmacists' knowledge and practice of issue related to usage of psychotropic medications in elderly people, in Gondar town Northwest, Ethiopia.

**Methods:**

A cross-sectional study was conducted among pharmacists working in community, health center, and hospital pharmacies in Gondar town from March 1 to May 30, 2020. A total of 73 medication retail outlets (40 pharmacies and 33 drug stores) were included in this study. Pharmacy personnel's knowledge and practice were assessed using self-administered validated questionnaires. Binary and multivariable logistic regression analyses were used to assess the association between different variables. *P* < 0.05 was used to declare the association.

**Result:**

A total of 144 pharmacists were included in the study; the mean age was 30.13 (SD ±5.87), ranging from 20 to 55years. The mean knowledge score was 7.789 (SD ±2.98), and 75 (52.1%) of them had poor knowledge. The mean practice score was 2.32 ± 0.912 (mean ± SD), and 77 (53.5%) of the respondents had poor practice. All participants had not taken on-the-job training about psychotropic medication. Work experience (*P* < 0.029) and personal monthly income (*P* < 0.046) were significantly associated with pharmacists' knowledge. There was a significant association between work experience and practice level (*P* < 0.043).

**Conclusion:**

The knowledge and practice of pharmacy personnel were low for issues related to the use of psychotropic medication in the elderly. This result indicates the need for training for pharmacists on pharmacotherapy of psychotropic medication.

## 1. Introduction

The elderly are defined by a chronological age of 65 years and older. This population has been and will be steadily increasing and expected to reach 22% in 2050 [1]. Aging involves progressive impairments in the functional reserve of many organs, which might also affect pharmacodynamics and pharmacokinetics of drugs [2, 3]. Elderly people are extremely prone to develop psychiatric morbidities due to aging of the brain, problems with physical health, cerebral pathologies, and a decrease in economic independence and breakdown of the family support system [4–7] because the use of psychotropic medications among elderly patients is high [8–11]. Psychopharmacology in elderly people is quite specific, and the tendency of older people to develop side effects to psychoactive medications is often due to physiological changes associated with aging, altered pharmacokinetics, and pharmacodynamics [12, 13]. The study report showed that the elderly patients on average took 2 to 5 drugs, and poly-pharmacy occurred in 20–50% of elderly patients [2, 3]. Consequently, adverse drug reactions and drug interactions are more common and more serious in the elderly [14, 15]. Frequent checks of used medications, particularly for drug interactions and use of medications, are considered appropriate for the elderly [12, 16].

Pharmacists are the most trusted and easily accessible health care practitioners and can play an indispensable role in selecting appropriate medicines, monitoring drug use, including adherence, optimal pharmaceutical care encouraging good health behaviors, and drug-related problems, which is a particular risk for the elderly. Pharmacists also provide appropriate information about interactions between drugs and other substances, including OTC drugs, dietary supplements, and foods [17–21]. Thus, to provide good healthcare services, pharmacists are expecting to have commitment, ethics, adequate knowledge, attitude, and skills of medication [22]. However, different studies have shown that pharmacists have low knowledge and practice related to medication use, including psychotropic medication, particularly in elderly patients [23, 24]. As in other developing countries, pharmacy practice in Ethiopia is evolving rapidly; however, there is a lack of empirical evidence about pharmacists' knowledge, practice, and experience of medication use [25]. In Ethiopia, nearly half of the pharmacists had an unfavorable attitude toward pharmaceutical care [24]. Thus, research is required to determine the magnitude of pharmacist knowledge and practice regarding psychotropic medication usage in the elderly.

Hence, to the best of the literature search done, no prior study has been conducted in Gondar town, Ethiopia, regarding assessing pharmacy personnel's knowledge and practice of issues related to psychotropic medication in the elderly. Thus, this study is aimed at assessing pharmacists' knowledge and practice of issue related to usage of psychotropic medications in elderly people and the associated factors in Gondar town, Ethiopia.

## 2. Method

### 2.1. Study Design and Setting

A cross-sectional study was conducted in Gondar town from March 1 to May 30, 2020. Gondar town is under the Amahara regional state of Ethiopia, which is located at 750 Km from Addis Ababa, the capital city of Ethiopia. According to United Nations-World Population Prospects, the population of Gondar in 2019 was 347,000. Gondar town has one compressive specialized hospital, two primary hospitals, and twelve health centers. There are 40 pharmacies and 33 drug stores currently serving people.

### 2.2. Inclusion Criteria and Exclusion Criteria

Pharmacy personnel with a diploma and above in pharmacy that works on the community, health center, and hospital pharmacy in Gondar town and willing to complete the study questionnaire were included. However, pharmacy personnel who had work experience of fewer than six months were excluded from the study.

### 2.3. Sample Size Determination and Sampling Techniques

All pharmacy professionals (155) that met the inclusion criteria were included in the study.

### 2.4. Study Variables

The dependent variables were pharmacists' knowledge and practice on psychotropic medication usage in the elderly. Independent variables included socioeconomic and demographic characteristics (age, gender, dress/residency, monthly income, marital status, living arrangements, level of education, and work experience) and respondent professional and environmental-related status (graduated collage, current working institution, and course and training towards psychotropic medication.

### 2.5. Data Collection Method

Data were collected using a structured self-administered questionnaire on 155 pharmacists who gave their consent for participation in the study. The data collection tool was developed after reviewing published literature [23, 26, 27] and consisted of 37 items that were divided into four sections. Questions 1−8 focused on the sociodemographic characteristics of the respondents, questions 9-13 focused on environmental-related status, and questions 14-32 and questions 33-37 are aimed at assessing the level of knowledge and practice of pharmacists toward psychotropic medication in the elderly, respectively. Respondents had to choose either “true,” “false,” or “I don't know” for knowledge questions and “Yes” or “No” for practice-related questions. Respondents will award one point for each correct answer and zero point for choosing wrong answer, “I do not know” is included to avoid guessing, and it counts zero point [28]. The final scores were calculated for the 19-statement knowledge test as the percentage correct, which ranged from 0% to 100%. Participants were considered to have “good knowledge” if they scored ≥50% (score of 10-19) and poor knowledge <50% (score of 0-9) [23].

### 2.6. Data Quality Assurance

The data collection tool was pretested on a 5% (8) pharmacy professional that was not included in the final analysis, and the questionnaire was sent to three senior pharmacists who were academicians and researchers for face validity, approval was obtained, and some modifications were done.

### 2.7. Data Analysis

The collected data were cleared, entered into, and analyzed using SPSS version 20.0 for Windows (SPSS Inc., Chicago, IL). The results are described in terms of frequencies, percentages, and means. The relationships among variables were analyzed using Pearson's chi-square test. Then, a bivariate analysis was carried out to evaluate the crude effect of each independent variable on the different study outcomes. Variables with a *P* value less than or equal to 0.2 in the bivariate analysis were included in multivariate logistic regression models. *P* < 0.05 was considered significant.

### 2.8. Ethical Clearance

Ethical clearance was taken from the Ethical Review Committee of the School of Pharmacy, College of Medicine and Health Sciences, University of Gondar, and verbal consent was obtained from the head of each pharmacy and participants.

### 2.9. Operational Definition

Good knowledge is the knowledge status of pharmacy personnel when they scored ≥50% [29]. Low knowledge is the knowledge status of pharmacy when they scored <50% [29]. Good practice is the practice status of pharmacy personnel when they score ≥mean. Low practice is the practice status of pharmacy personnel when they score <mean.

## 3. Result

### 3.1. Sociodemographic and Professional-Related Characteristics of the Respondents

Of the 155 questionnaires, 144 were adequately filled and returned, resulting in a response rate of 92.9%. Among the 144 respondents, 79 (54.9%) were male. The mean age of the respondents was 30.13 ± 5.87 (mean ± SD), ranging from 20 to 55years; 73 (50.7%) of participants were in the age group 18-30 years old, and 77 (53.5%) of the respondents were married. Majority, 89 (61.8%), of the respondents were living with family or relative, 77 (53.5%) respondents were pharmacy technicians and 76 (52.8%) and 78 (54.2%) of the respondents had less than 5 years of professional work experience and less than 5 thousand birr monthly income, respectively (presented in Table 1).

### 3.2. Environmental-Related Status

A majority of 83 (57.6%) respondents were graduated from government institutions, and 82 (56.9%) of the respondents worked on government institutions. Regarding the practice setting, 61 (42.4%) of the respondents worked on community pharmacies. Moreover, all respondents were taking a course on psychotropic medication during the pharmacy learning program, and all of them had not taken on-the-job training about psychotropic medication (summarized in Table 2).

### 3.3. Pharmacist's Knowledge of Psychotropic Medication Usage in Elderly Patients

One hundred two (70.8%) of the respondents knew that the dose of antipsychotics or benzodiazepines should be reduced in elderly people due to altered metabolism rates and higher sensitivity. 35 (24.3%) respondents response that low-dose hypnosedative medications given over the short term might reverse the physiological changes in sleep patterns in elderly people. And 55 (38.2%) knew the recommended daily dose of risperidone.

Regarding pharmacists' knowledge about the selection of appropriate medications, 66 (45.8%) respondents knew the preferences of antipsychotics over benzodiazepine for sedating elderly patients with severe agitation or delirium. In addition, 58 (40.3%) of them knew that antipsychotic medications reduce symptoms such as delusions and hallucinations.

Regarding knowledge about side effects, 73 (50.7%) respondents knew that the use of hypnosedative can lead to physical and emotional dependence. Only 56 (38.9%) of them knew long-term (3 months or above) intake of antipsychotic medications increases the risk of cerebrovascular accidents. Half of the respondents (72 (50%) of them) knew that benzodiazepines can lead to side effects in the old like confusion, memory, and concentration disorders, as shown in Table 3.

**Figure 1:**
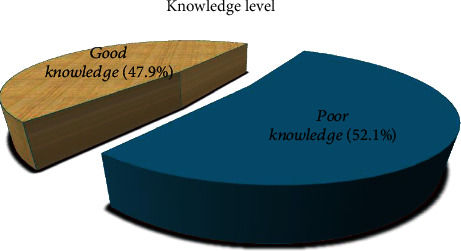
Pharmacy personnel knowledge level on psychotropic medication used in elderly people in Gondar, Ethiopia, April 2020, *N* = 144.

Regarding the knowledge level of respondents, the overall knowledge score of the respondents ranged from 0 to 19, with a mean knowledge score of 7.789 (SD = 2.98) out of the 19 knowledge questions. 75 (52.1%) of them had poor knowledge, while 69 (47.9%) of them had good knowledge levels, as presented in Figure 1.

**Figure 2:**
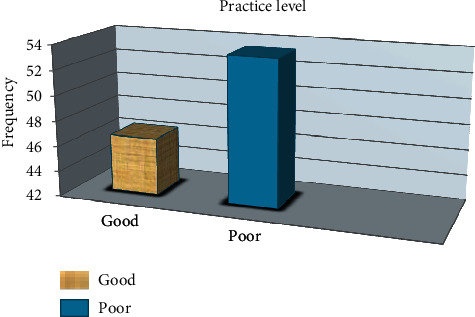
Practice level of pharmacy personnel toward psychotropic medication used in elderly people in Gondar, Ethiopia, April 2020, *N* = 144.

The mean score for level of practice was 2.32 ± 0.912 (mean ± SD) out of 5 practice questions. 77 (53.5%) of the respondents had poor practice. Study participants who scored less than the mean value were regarded as low practice, whereas participants who scored more than the mean value were regarded as good practice (presented in Figure 2).

**Figure 3:**
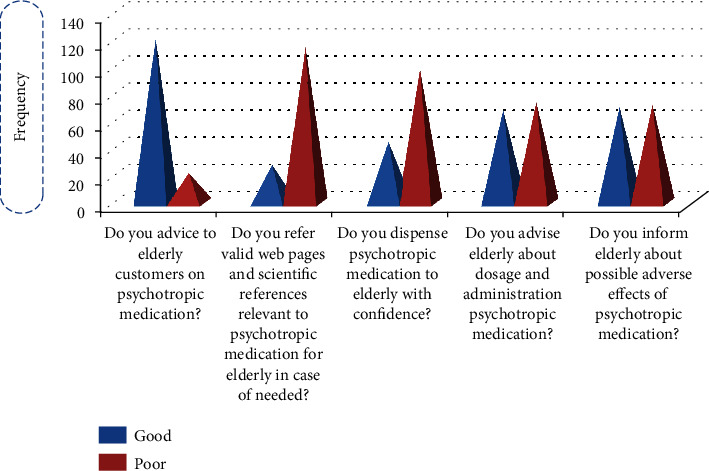
Practice-related response frequency of pharmacy personnel toward psychotropic medication used in elderly people in Gondar, Ethiopia, April 2020, *N* = 144.

122 (84.7%) respondents advice to elderly customers on psychotropic medication, but only 45 (31.3%) of them dispense psychotropic medication to the elderly with confidence. In the use of valid reference, 28 (19.4%) pharmacists practice when it is necessary. In addition, 69 (47.9%) of the respondents advise the elderly about the dosage and administration of psychotropic medications (presented in Figure 3).

### 3.4. Association between Knowledge and Sociodemographic Characteristics

Accordingly, in the multivariate logistic analysis, pharmacy personnel who had work experiences greater than or equal to 5 years were 4.1 times more likely to have good knowledge than those who had less than 5 years of work experience (AOR, 4.173; 95% CI, 1.156-15.062; *P* < 0.029). Pharmacy personnel with a monthly income greater than 5000 birr were 2.7 times more likely to have good knowledge than those with a monthly income less than 5000 birr (AOR, 2.763; 95% CI, 1.020-7.487; *P* < 0.046) (summarized in Table 4.

### 3.5. Association between Practice and Sociodemographic Characteristics

Table 5 indicates that there was a statistically significant association between work experiences and practice level (AOR, 3.725; 95% CI, 1.040-13.349; *P* < 0.043). Concerning age, marital status, practice setting, and other sociodemographic characteristics were not significantly associated.

## 4. Discussion

Medication use during the elderly period is common, and prevalence continues to increase [30, 31]. Pharmacy professionals must carefully appraise the potential risks of medication use versus risks for the elderly. They should provide patients with information regarding both benefits and risks of medication use while also discussing the limitations of available knowledge so that the elderly will be empowered to make informed decisions that are best for them. Pharmacy personnel have great potential to modify and optimize drug therapy in the elderly [32].

The findings of this study showed that 75 (52.1%) of the respondents had poor knowledge. This finding was of comparably higher values than the study conducted in Palestine (37.93%) [23]. This difference might be due to curriculum differences in the country and gaps in on-the-job training. This poor knowledge could be attributed to the fact that participants did not receive any training about psychotropic medication. This fact highlights the importance of on-the-job training of pharmacists and emphasizes the role of the Ministry of Health in improving the performance of pharmacists and expanding their role in managing patients beyond dispensing medications through continuing education. On the other hand, the finding of this study is lower than study done on antiepileptic medication in Khartoum, Sudan, 85.3% [29].

Concerning the knowledge of pharmacy personnel on the dosage of psychotropic medications (Table 3, statements 1–4), 102 (70.8%) of the pharmacists knew that the dose of antipsychotics or benzodiazepines should be reduced in elderly people due to altered metabolism rates and higher sensitivity. Of these, 55 (38.2%) knew the recommended daily dose of risperidone in elderly people with severe behavioral disorders in dementia, and also, 35 (24.3%) of them knew that low-dose hypnosedative medications given over the short term might reverse the physiological changes in sleep patterns in elderly people. However, only 49 (34%) correctly answered the statement on the recommended daily dosage of olanzapine in older people with severe behavioral disorders in dementia. In general, in this study, participants gave less correct answers than in the study done in Belgium [33]. This might be due to differences in profession and acquired training.

The findings of this study showed that none of the respondents had attended training about psychotropic medication. Recently, it has been reported that practical training programs increased learning motivation among pharmacy students [34]. Incorporating practical sessions in pharmacology and pharmacotherapy might have positive effects on pharmacists' knowledge, attitudes, and skills [35]. Similarly, using computer simulations have been shown to enhance knowledge of medications and pharmacology [36].

In the present study, the selection of appropriate psychotropic medications (Table 3, statements 5–11) has an average score of 39.08%. Again, the pharmacy personnel in this study performed worse than Belgian nurses [33]. This may be due to sociodemographic and environment-related differences in respondent and curriculum differences between countries. Pharmacy as a profession is growing. Pharmacists are often consulted by physicians in selecting the most appropriate medications for patients [37]. Therefore, pharmacists' knowledge should be optimized. Failing to do this might have severe negative consequences on the health care system. Many pharmacy schools have realized this and revised their pharmacy curricula accordingly in an attempt to acquaint pharmacy graduates with an optimal level of knowledge [38].

This finding showed that the performance of pharmacy personnel on statements related to side effects (Table 3, statements 12-19) was relatively better than a statement related to dosage and selection of appropriate psychotropic medication.

Of the pharmacists, 73 (50.7%) knew that hypnosedatives can lead to physical and emotional dependence; 62 (43.1%) knew that antipsychotics are associated with anticholinergic side effects; 59 (41%) knew that atypical antipsychotics can lead to weight gain; 72 (50%) knew that benzodiazepines have side effects in the elderly like confusion, memory, and concentration disorders; 60 (41.7%) knew that haloperidol was associated with akathisia, and antipsychotics increased the prevalence of falls; 47 (32.6%) knew that antipsychotics cause postural hypotension; and 56 (38.9%) knew that antipsychotics increased the risk of cerebrovascular accidents. The finding of this study has lower value (42.37%) than that of the study done by Belgium nurses (56.57%) [33]. This might be due to the fact that our country has a low-quality controlling system in pharmacy personnel, and less concern is given by the government to improve knowledge of pharmacy personnel. However, there is a higher value with a study done by American community pharmacists regarding donepezil adverse effects [39]. Despite gaps in knowledge, as experts on medications, pharmacists need to be knowledgeable about side effects and drug-drug interactions, including those of psychotropic medications. If well acquainted, pharmacists can play a prominent role in resolving medication-related problems [40–42]. Pharmacists' inability to alert patients of the potential side effects of psychotropic medications might severely impact the health of the patients [43, 44].

In the present study, about 122 (84.7%) claimed that they advise elderly customers on psychotropic medication. 28 (19.4%) of them used reference to psychotropic medication, 45 (31.3%) of them dispense with confidence, and 69 (47.9) and 71 (49.3) of them advise the elderly about the dosage and inform the elderly about the possible adverse effects, respectively. In general, from the findings of this study, 77 (53.5%) of them had a poor level of practice. This might be due to their knowledge level. As we can see in Figure 1, 75 (52.1%) of them had poor knowledge, which may have led to their poor practice. This finding was in agreement with another study conducted on dietary supplement knowledge, attitude, and practice of pharmacists in Tehran, which revealed that majority of them had poor practice, and there were positive and significant relationships between level of practice and knowledge [26].

In the present study, pharmacy personnel having work experience ≥5 years (AOR, 4.173; 95% CI, 1.156-15.062 and AOR, 3.725; 95% CI, 1.040-13.349) were significantly associated with knowledge and practice level, respectively. This may be because as the number of experiences increases the chance of working with different teams, learning from personal mistakes, and increasing practical skills, these have a positive impact on knowledge level. This finding was in agreement with previous studies done on medication use (*P* < 0.001) in Utha and Qatar [45, 46]. However, a study conducted on antiepileptic medication in Palestinian pharmacists reported that there was no significant association (*P* = 0.249) between work experience and level of knowledge [28].

In this study, respondents with monthly income >5000 birr were significantly associated (AOR, 2.763; 95% CI, 1.020-7.487) with their knowledge level than having ≤5000 birr personal monthly income. The majority of those getting ≥5000 birr per month might have a higher chance of getting information from social media than a person getting ≤5000 birr. However, contrary to our findings, the study done in Mekele, Ethiopia [47], showed that no significant association between monthly income and knowledge (AOR, 4.411; 95% CI 0.577-33.712) on generic medication knowledge of pharmacy personnel practicing in Mekele [47].

### 4.1. Strengths and Limitations of the Study

To the best of the literature search done, this is the first study to assess pharmacists' knowledge and practice regarding issues related to usage of psychotropic medication in elderly people in Ethiopia, so this study can provide baseline information.

However, there were some limitations with the present study. The possible limitation is that the sample size is small to give representative and powered data on associated factors for pharmacist's knowledge and practice, and this is conducted only in Gondar town. Therefore, studies should be conducted with a large sample size at multiple sites. The use of this tool could have underestimated or overestimated the knowledge of pharmacists, as their performance could have been different if the test was based on multiple-choice questions. The test included questions in the form of statements. The respondents' performance could have been different if case-based scenarios were included in the test. The study was assessed using only a quantitative method. Moreover, this is a cross-sectional study design, whereby claims about the directionality of a causal relationship between the dependent and independent variables cannot be verified.

## 5. Conclusion

The knowledge and practice of pharmacy personnel were low towards issues related to the use of psychotropic medication in the elderly. This study revealed that knowledge of pharmacy personnel regarding issues related to the use of psychotropic medication in the elderly was strongly associated with work experience and personal monthly income.

In addition, this practice is also associated with work experience. Further investigation is needed to reveal the reasons which led to this knowledge gap. This issue needs attention from the government in order to provide optimal care. This gap can be improved by providing education and training to practicing pharmacy personnel by organizing continuing medical education programs focused on pharmacotherapy of psychotropic medication.

## Figures and Tables

**Table 1:** Sociodemographic characteristics of pharmacy personnel in Gondar town, Ethiopia, April 2020, *N* = 144.

Variable	Frequency (%)
Age	
18-30	73 (50.7)
30-40	65 (45.1)
>40	6 (4.2)
Gender	
Male	79 (54.9)
Female	65 (45.1)
Residence	
Urban	127 (88.2)
Rural	17 (11.8)
Marital status	
Single	67 (46.5)
Married	77 (53.5)
Living arrangement	
Alone	55 (38.2)
With family or relative	89 (61.8)
Level of education	
Pharmacy technician	77 (53.5)
Degree and above in pharmacy	67 (46.5)
Work experience	
<5 years	76 (52.8)
≥5 years	68 (47.2)
Personal monthly income (birr)	
<5000	78 (54.2)
≥5000	66 (45.8)

**Table 2:** Pharmacy personnel's professional and environmental-related status in Gondar town, Ethiopia, April 2020, *N* = 144.

Variable	Frequency (%)
Graduated college	
Government	83 (57.6)
Private	61 (42.4)
Current working institution	
Government	82 (56.9)
Private	62 (43.1)
Practice setting	
Hospital	60 (41.7)
Health center	23 (16)
Community pharmacy	61 (42.4)
Had you take a course on psychotropic medication during pharmacy learning program	
Yes	144 (100)
No	0
Have you ever take training about psychotropic medication after graduation	
Yes	144 (100)
No	0

**Table 3:** Pharmacy personnel's knowledge on psychotropic medication used in elderly people, Gondar, Ethiopia, April 2020, *N* = 144.

Statement		Response		
		True	False	I do not know
Statement related to dosage				
1	Due to changed metabolism and a higher sensitivity, older persons need a lower dose of antipsychotics or benzodiazepines in order to get the same effect.	**102 (70.8)**	23 (16)	19 (13.2)
2	The recommended daily dosage of olanzapine is 50 to 100 mg in older people with severe behavioral disorders in dementia.	83 (57.6)	**49 (34)**	12 (8.3)
3	The recommended daily dose of risperidone is 0.5 to 2 mg in older people with severe behavioral disorders in dementia.	**55 (38.2)**	30 (20.8)	59 (41)
4	Ageing is associated with physiological changes in the sleeping pattern. To reverse these changes in the sleep pattern, hypnosedative medications may be used in low dose during a short period.	**35 (24.3)**	41 (28.5)	68 (47.2)
Statements related to selection of appropriate medications				
5	With the exception of delirium tremens, antipsychotics are preferred above benzodiazepines for sedating older patients with severe agitation or delirium.	**66 (45.8)**	34 (23.6)	44 (30.6)
6	Antipsychotic medications can have a place in the treatment of delirium.	**60 (41.7)**	24 (16.7)	60 (41.7)
7	Antipsychotic medications reduce such symptoms as delusions and hallucinations.	**58 (40.3)**	23 (16)	63 (43.8)
8	In the care of older adults with dementia, antipsychotic medications are preferred over behavior-oriented therapy.	**50 (34.7)**	24 (16.7)	70 (48.6)
9	Only in severe cases of sleeplessness and failure of alternative therapies with proven effectiveness, hypnosedatives can be administrated for a short period of time in the old.	**54 (37.5)**	22 (15.3)	68 (47.2)
10	The effects of diazepam, a benzodiazepine, can last for a long time, making it not proper to use in this age category.	**51 (35.4)**	54 (37.5)	39 (27.1)
11	Next to nonpharmacological therapies, hypnosedatives are to be used for treatment and minimization of the symptoms of anxiety disorders.	**55 (38.2)**	43 (29.9)	46 (31.9)
Statement related to side effect				
12	Use of hypnosedatives can lead to physical and emotional dependency.	**73 (50.7)**	10 (6.9)	61 (42.4)
13	Long-term (3 months or above) intake of antipsychotic medications increases the risk for cerebrovascular accidents	**56 (38.9)**	22 (15.3)	66 (45.8)
14	Antipsychotic medications can cause side effects in the old such as disorientation, urine retention, dry mouth, and blurred vision.	**62 (43.1)**	19 (13.2)	63 (43.8)
15	Long-term intake (3 months or above) of atypical antipsychotic medications can lead to an increase in weight.	**59 (41)**	30 (20.8)	55 (38.2)
16	Patients starting on antipsychotic medication are susceptible to postural hypotension.	**47 (32.6)**	36 (25)	61 (42.4)
17	One of the side effects of haloperidol is akathisia, which manifests with constant pacing and restlessness.	**60 (41.7)**	19 (13.2)	65 (45.1)
18	There is a connection between long-term (3 months or above) intake of antipsychotic medications and the prevalence of falls in the old.	**59 (41)**	22 (15.3)	63 (43.8)
19	Benzodiazepines can lead to side effects in the old like confusion, memory, and concentration disorders.	**72 (50)**	16 (11.1)	56 (38.9)

Values are represented as numbers (%), and correct answers are in boldface.

**Table 4:** Association between sociodemographic characteristics and knowledge level of pharmacy personnel toward psychotropic medication used in elderly people, Gondar, Ethiopia, April 2020, *N* = 144.

Variable	Total knowledge score				
	Good knowledge	Poor knowledge	COR (95% CI)	AOR (95% CI)	*P* value
Age					0.245
<30	28	45	1^∗∗^	1^∗∗^	
30-40	38	27	1.607 (0.303-8.524)	0.249 (0.28-2.217)	
>40	3	3	0.711 (0.133-3.792)	0.700 (0.104-4.694)	
Marital status					0.348
Single	23	44	1^∗∗^	1^∗∗^	
Married	46	31	2.839 (1.439-5.601)	1.493 (0.647-3.447)	
Level of education					0.656
Pharmacy technician	27	50	1^∗∗^	1^∗∗^	
B. Pharm	42	25	3.111 (1.574-6.149)	1.245 (0.475-3.268)	
Graduated college					0.555
Government	48	35	1^∗∗^	1^∗∗^	
Private	21	40	0.383 (0.193-0.759)	0.775 (0.332-1.809)	
Work experience					0.029^∗^
<5 years	25	51	1^∗∗^	1^∗∗^	
≥5 years	44	24	3.740 (1.876-7.456)	4.173 (1.156-15.062)	
Monthly income (birr)					0.046^∗^
<5000	24	54	1^∗∗^	1^∗∗^	
≥5000	45	21	4.821 (2.378-9.775)	2.763 (1.020-7.487)	
Practice setting					0.133
Hospital	36	24	3.429 (1.227-9.579)	2.750 (0.850-8.897)	
Health center	7	16	2.019 (0.979-4.165)	0.857 (0.336-2.184)	
Community pharmacy	26	35	1^∗∗^	1^∗∗^	

^∗∗^Constant, ^∗^significant.

**Table 5:** Association between sociodemographic characteristics and practice level of pharmacy personnel toward psychotropic medication used in elderly people, Gondar, Ethiopia, April 2020, *N* = 144.

Variable	Total practice score				
	GP	PP	COR (95% CI)	AOR (95% CI)	*P* value
Age					0.127
<30	29	44	1^∗∗^	1^∗∗^	
30-40	36	29	0.759 (0.130-4.414)	0.123 (0.013-1.141)	
>40	2	4	0.403 (0.69-2.356)	0.362 (0.052-2.509)	
Marital status					0.805
Single	26	41	1^∗∗^	1^∗∗^	
Married	41	36	1.796 (1.024-3.882)	0.898 (0.381-2.115)	
Level of education					0.210
Pharmacy technician	26	52	1^∗∗^	1^∗∗^	
B. Pharm	41	26	3.093 (1.565-6.115)	1.836 (0.710-4.744)	
Graduated college					0.058
Government	49	34	1^∗∗^	1^∗∗^	
Private	18	43	0.290 (0.144-0.587)	0.448 (0.195-1.028)	
Work experience					0.043^∗^
<5 years	26	50	1^∗∗^	1^∗∗^	
≥5 years	41	27	2.920 (1.481-5.756)	3.725 (1.040-13.349)	
Monthly income (birr)					0.359
<5000	26	52	1^∗∗^	1^∗∗^	
≥5000	41	25	3.280 (1.654-6.506)	1.599 (0.587-4.361)	
Current working institution					0.632
Government	42	40	1^∗∗^	1^∗∗^	
Private	25	37	0.644 (0.330-1.254)	0.519 (0.035-7.596)	
Practice setting					0.259
Hospital	35	25	3.200 (1.147-8.926)	2.466 (0.780-7.792)	
Health center	7	16	2.016 (0.978-4.157)	0.489 (0.033-7.202)	
Community pharmacy	25	36	1^∗∗^	1^∗∗^	

^∗∗^Constant; ^∗^significant; GP: good practice; PP: poor practice.

## Data Availability

The data used to support the findings of this study are available from the corresponding author upon request.
